# Kinetic and thermodynamic study on the esterification of oleic acid over SO_3_H-functionalized eucalyptus tree bark biochar catalyst

**DOI:** 10.1038/s41598-022-12539-0

**Published:** 2022-05-23

**Authors:** Adeyinka Sikiru Yusuff

**Affiliations:** Department of Chemical and Petroleum Engineering, College of Engineering, Afe Babalola University, Ado-Ekiti, Nigeria

**Keywords:** Chemical engineering, Green chemistry

## Abstract

Herein, esterification of oleic acid (OA) over tosylic acid functionalized eucalyptus bark biochar (TsOH-MBC) to synthesize fatty acid methyl ester (FAME) was investigated. The TsOH-MBC catalyst was prepared via pyrolysis-activation-sulfonation process at various impregnation ratios and was characterized by SEM, FTIR, EDX, XRD, BET, TGA and acid site density techniques. The catalytic performance of the sulfonated biochar catalyst was described in terms of acidity and FAME yield. 6 g of sulfonic acid loaded on 10 g of MBC (6TsOH-MBC) appeared to be most appropriate combination to achieve a highly active catalyst for the esterification of OA with 96.28% conversion to FAME at 80 °C for 5 h with catalyst loading of 4.0 wt% and 8:1 methanol/OA molar ratio. The catalytic reaction kinetic data were very well described by the second-order model, with a rate coefficient of 0.223 mL mol^−1^ h^−1^ at 80 °C and activation energy of 81.77 kJ mol^−1^. The thermodynamic parameters such as $\Delta H$, $\Delta S$ and $\Delta G$ were determined to be 78.94 kJ mol^−1^, 135.3 J mol^−1^ K^−1^ and 33.03 kJ mol^−1^, respectively. This research provided an environmentally friendly procedure for FAME production that could be replicated on a commercial scale.

## Introduction

Fatty acid methyl ester (FAME), also known as biodiesel, can be produced by esterifying fatty acids with an acid catalyst or transesterifying triglycerides with a base or acid catalyst. Esterification via acid catalysis is the preferred method of producing FAME as base catalysis often results in side reaction (saponification) which reduces product yield and quality^1^. The most commonly used catalysts for fatty acid esterification are homogeneous catalysts such as H_2_SO_4_, HCl, and HF. These catalysts have limitations and this has made the cost of FAME produced by using them very expensive. For instance, reusability of spent catalyst is impossible because the liquid catalyst mixes with reactants, resulting in wastewater generation as product washing is required to remove the dissolved catalyst^2,3^. However, FAME production via heterogeneous acid catalyzed esterification could reduce the occurrence of corrosion, ensure a facile and an ecofriendly process, and guarantee catalyst reusability^4^. Furthermore, the physicochemical properties of the heterogeneous acid catalyst could be easily modified to improve catalytic activity and stability^3,5^.

In recent times, several heterogeneous acid catalysts, including H_2_SO_4_/activated carbon^1^, H_2_SO_4_/char^3,4^, H_2_SO_4_/ZrO_2_^6,7^, Al_2_O_3_/ZrO_2_^8^ and heteropoly acid/metal–organic framework^9^ have been synthesized and used in catalyzing esterification reaction for FAME production. Amongst the numerous solid acid catalysts available, acid (sulfonated) modified carbon-based catalysts have been proposed as environmentally friendly, low cost and green catalyst due to its ability to be derived from waste or biomass^1^. To address the issue, eucalyptus tree bark, which contains approximately 84% organic and 16% inorganic matter, is widely available and can be used as a source of biochar-based catalyst^10^, which in turn could address the environmental problem posed by littered eucalyptus tree bark.

Biochar is a carbon-rich material produced through the pyrolysis of organic matter at high temperatures (typically 300–650 °C) with little or no oxygen to prevent combustion^11,12^. Previous research has shown that biochar, as a catalyst support, is effective in fatty acid esterification due to its good textural properties, abundant active surface functional groups and high adsorption capacity^1,13^. When comparing commercially produced activated charcoal to biochar, however, it is necessary to improve the latter's physicochemical properties. The chemical treatment of biochar with zinc chloride (ZnCl_2_) as a modifying agent can result in activated biochar with a hydrophobic surface, a high surface area, a well-developed porous structure, and an abundance of surface functional groups^1,13^. Yusuff et al.^14^ discovered that activating poultry litter biochar with ZnCl_2_ produced an adsorbent with a high sorption capacity for crystal violet removal from aqueous solution. Thus, ZnCl_2_-activated biochar was considered as a structure support for the development of a heterogeneous acid catalyst.

It has been reported that the synthesis of SO_3_H (sulfonic acid group) functionalized carbon based material via sulfonation using H_2_SO_4_ produces a catalyst with high acidic strength and better activity^1,15,16^. Dechakhumwat et al.^3^ reported that a solid acid catalyst prepared by impregnating corncob biochar with H_2_SO_4_ increased the yield of esterified product. However, using H_2_SO_4_ as a sulfonating agent for sulfonated catalyst development can cause corrosion if a high pressure withstanding reactor is not used, necessitating special precautions^11,17^. Thus, there is a need to find alternative sulfonating agents that are less corrosive, have a low vapour pressure and have better catalytic activity. As a result, tosylic acid (TsOH) or p-Toluenesulfonic acid (p-TSA) has been discovered to be a suitable replacement for H_2_SO_4_ because the former possesses the aforementioned properties^3,18^. Russo et al.^19^ used a SO_3_H-functionalized C/SiO_2_ composite obtained through TsOH and H_2_SO_4_ sulfonation for biomass conversion and discovered that TsOH prevented damaging corrosion.

Several studies on the kinetics of fatty acid esterification over various catalysts have been conducted. Many of the reported studies advocated for the use of a pseudo-first-order kinetic model to evaluate the kinetic parameters^20–24^. A kinetic study with a second-order model for esterification of oleic acid over zinc acetate catalyst had been reported, and it agreed reasonably well with the experimental data obtained^25^. Nonetheless, there is a scarcity of information on the kinetics and thermodynamic analysis of oleic acid esterification over a biomass-derived solid acid catalyst.

The current research focused on the synthesis of a SO_3_H-functionalized eucalyptus tree bark biochar catalyst for oleic acid esterification. To investigate the physicochemical properties of the as-prepared catalyst, several characterization techniques (SEM, FTIR, EDX, XRD, TGA, BET and acidity) were used. Kinetics and thermodynamic analyses of the catalytic reaction process were performed using different model equations. In addition, the stability test was carried out under optimal reaction conditions to determine the possibility of catalyst reuse.

## Materials and methods

### Materials

Eucalyptus tree shed barks (ETBs) were collected from the garden waste bins at Afe Babalola University, Ado-Ekiti, Nigeria. Tosylic acid (TsOH, $\ge$ 98.0%, Merck), oleic acid (65–88%, Merck), methanol (99.8%), n-hexane (99%), methyl heptadecanoate (99.5%), zinc chloride (98%) and hydrochloric acid (36%) were all purchased from Sigma-Aldrich Chemical Industries, India.

### Catalyst preparation

#### Preparation of ZnCl_2_ modified biochar

Firstly, the collected ETB was thoroughly washed with clean water to remove sand, dried at 80 °C for 6 h in an oven, and then ground into powder using mortar and pestle. After that, ETB powder was sieved through 0.3 mm sieve mesh to achieve the needed particle size. To make biochar, the sieved ETB powder was pyrolyzed in a muffle furnace with no oxygen (at 500 °C for 1.5 h and a ramping rate of 5 °C/min). The pyrolyzed material (biochar, BC) was allowed to cool before being stored in a glass container. To activate the biochar, 10.0 g of BC powder was mixed with 200 mL of 3 M ZnCl_2_ solution in a beaker, stirred on a magnetic stirrer (for 5 h at 60 °C) and filtered through filter paper. The activated biochar was then washed several times with distilled water to remove impurities until the pH of the solution reached neutral. After that, the sample was dried at 110 °C overnight. The obtained chemically activated biochars are henceforth referred to as ZnCl_2_-modified biochar (MBC).

#### Preparation of SO_*3*_H-functionalized eucalyptus tree bark biochar catalyst

A fixed amount of the MBC sample (10 g) was suspended in distilled water, and different amounts of TsOH (2, 4, 6, and 8 g) were gently added and stirred for 5 h at 60 °C on a magnetic stirrer. The resulting homogenized solution was then oven dried for 24 h at 100 °C. The dried sulfonated biochar catalysts were denoted as mTsOH-MBC, where m denotes the mass of TsOH used for MBC sulfonation, e.g., 2TsOH-MBC denoted that 2 g of TsOH was used for the sulfonation process.

### Analysis of MBC and TsOH-MBC catalyst

The surface functional groups, phase composition and crystallographic structure, thermal decomposition trend, surface morphology and elemental composition and textural characteristics of the MBC and optimal mTsOH-MBC catalyst were examined by using Fourier transform infrared (FTIR) spectrometer (Perkin-Elmer Spectrum TWO, spectra range 4000–400 cm^−1^), powder X-ray diffractometer (XRD) (D8 Advance, Bruker AXS Gmbh, Germany), TGA/DTG analyzer (Shimadzu DTG60, Japan; analysis condition: N_2_ carrier flow rate = 100 mL min^−1^, heating rate = 15 °C min^−1^ and range of temperature = 30–900 °C), field-emission scanning electron microscope (FE-SEM, Quanta 200F, Eindhoven, Netherland), Micromeritics model analyzer (ASAP 2010, USA; analysis condition: the sample was degassed at 250 °C for 5 h to remove adsorbed molecules from the surface, and Brunauer–Emmett–Teller (BET) surface measurement was done under N_2_ gas at 77 K), respectively. Moreover, acid density (or acidity) of the optimal sulfonated biochar catalyst was determined using ion-exchange titration procedures reported by Nda-Umar et al.^11^. For the determination of the property, 0.05 g of the optimal mTsOH-MBC catalyst sample was suspended in 20 mL of 2 M NaCl solution and mixed for 1 h on a magnetic stirrer. The suspension was thereafter filtered, and the filtrate obtained was titrated against 0.05 M NaOH using phenolphthalein as an indicator. The acidity, expressed as mmol of H^+^/g cat, was estimated using Eq. (1).

1 $${A}_{d}= \frac{{V}_{NaOH }\times {C}_{NaOH}}{{M}_{c}},$$

where ${A}_{d}$ is the acidity (mmol of H^+^/g cat), ${V}_{NaOH}$ is the volume of NaOH consumed and ${M}_{c}$ is the mass of catalyst used.

### Catalytic activity studies

#### Esterification of oleic acid

Esterification of oleic acid (OA) with methanol over TsOH-MBC catalyst was conducted in a 100 mL two-neck round bottom flask connected with a reflux condenser and a magnetic stirrer. Throughout the catalytic reaction process, the flask was inserted in a silicon oil bath to control the temperature. For each experiment, a certain mass of the catalyst sample was added to methanol, mixed for 10 min, and 10 g of OA was added to the mixture. After that, the reaction mixture was heated to the desired reaction temperature and continuous stirring began immediately at 500 rpm in order to avoid mass transfer limitation. The reaction process was carried out at 60 °C for 2 h with methanol/OA molar ratio of 8:1 using 2 wt% catalyst loading. Following the completion of the reaction, the reaction products were centrifuged at 7500 rpm for 10 min to facilitate the removal of spent catalyst. To remove unreacted methanol and water, the centrifuged liquid was evaporated using a rotary evaporator at 105 °C.

#### Esterified product analysis

The methyl ester content in the evaporated product was analyzed by gas chromatographic device (Agilent GC, 7890A, USA) coupled with flame ionization detector (FID) (GC-FID) and a capillary column (J & W DH-5HT; dimension: 15 m $\times$ 0.32 mm $\times$ 0.1 μL). Helium and methyl heptadecanoate were used as carrier gas and internal standard, respectively. The operating condition of the GC-FID was maintained at 350 °C and 30 °C min^−1^ for 10 min. The FAME yield (Y) and conversion to FAME (X) were calculated using Eqs. (2) and (3), respectively.

2 $$Y= \frac{\left(\sum A \right)- {A}_{I} }{{A}_{I}} \times \frac{{C}_{I }\times {V}_{I}}{m} \times 100\%,$$

3 $$X= \frac{\left(\sum A \right)- {A}_{I} }{{A}_{I}} \times \frac{{C}_{I }\times {V}_{I}}{m} \times \frac{{M}_{F}}{{M}_{OA}} \times 100\%,$$

where $\sum A$ indicates overall peak areas of FAME from C_14_ to C_22_, A_I_ is the peak area associated with the internal standard, V_I_ and C_I_ are the volume and concentration of internal standard, respectively, m is the sample weight, ${M}_{F}$ is the mass of FAME obtained and ${M}_{OA}$ is the mass of oleic acid used.

### Kinetics and thermodynamic studies

The kinetics and thermodynamic behaviours of OA esterification over 6TsOH-MBC catalyst was studied at different values of temperature (55, 65 and 80 °C) and time (1–5 h). The kinetics and thermodynamic parameters were evaluated as explained below:

#### Investigation of the reaction parameters

The strategy used in estimating the esterification reaction parameters (rate constant and reaction order) has been previously reported by Toor et al.^26^. Esterification of OA with methanol, a reversible catalytic reaction process, is illustrated stoichiometrically as follows:

4 $${C}_{17}{H}_{33}COOH \left(OA\right)+ {CH}_{3}OH\left(Methanol\right)\leftrightarrow {C}_{17}{H}_{33}COOC{H}_{3 }\left(methyl \; oleate\right)+ {H}_{2}O \left(water\right).$$

Because the amount of methanol (M) used exceeded the stoichiometric amount, the irreversible non-elementary reaction rate equation is as follows:

5 $$-\frac{d{C}_{OA}}{dt}=k{C}_{OA}^{\alpha }{C}_{M}^{\beta }.$$

Expressing Eq. (5) in terms of conversion, we have

6a $${C}_{OA}= {C}_{{OA}_{o}}(1- {X}_{OA})$$

6b $${C}_{M}= {C}_{{OA}_{o}}(\varnothing - {X}_{OA})$$

6c $$\varnothing = \frac{{C}_{{M}_{o}}}{{C}_{{OA}_{o}}}$$

Inserting Eqs. (6a–c) in Eq. (5) and rearranging, we have:

7 $$\frac{d{X}_{OA}}{dt}=k{C}_{{OA}_{o}}^{\left(\alpha + \beta -1\right)}{(1- {X}_{OA})}^{\alpha }{(\varnothing - {X}_{OA})}^{\beta },$$

where ${C}_{{OA}_{o}}$, ${C}_{{M}_{o}}$ t, $k$, $\alpha$, $\beta$, ${C}_{OA}$, ${C}_{M}$, ${X}_{OA}$, $\varnothing$ are initial concentration of oleic acid (mol mL^−1^), initial concentration of methanol (mol mL^−1^), reaction time (h), rate constant (unit depends on reaction order), reaction order of oleic acid, reaction order of methanol, final concentration of oleic acid, final concentration of methanol, fractional conversion of oleic acid and methanol/oleic acid molar ratio.

Since many studies on esterification of organic acid with alcohol reported either zero or first order^24,25^, it was presumed in this study that the order of reaction between OA and methanol could be pseudo-first or second-order, thus indicating that the overall reaction order must be equal to or less than two ($\alpha + \beta \le 2$).

Case 1: Assuming pseudo-first-order reaction rate equation by considering $\alpha$ = 1 and $\beta$ = 0, thus Eq. (7) upon integration becomes:

8 $$-In\left(1- {X}_{OA}\right)= {k}_{1}t,$$

where ${k}_{1}$ is the rate constant for first-order reaction (h^−1^)

Case 2: A second-order reaction rate was considered by assuming that $\alpha$ = 1 and $\beta$ = 1. Therefore, Eq. (7) upon resolution by partial fraction, integration and rearrangement becomes:

9 $$In\left(\frac{\left(\varnothing - {X}_{OA}\right)}{\varnothing \left(1- {X}_{OA}\right)}\right)= {C}_{{OA}_{o}}\left(\varnothing -1\right){k}_{2}t,$$

where ${k}_{2}$ is the rate constant for second-order reaction (mL mol^−1^ h^−1^).

#### Estimation of thermodynamic properties

By fitting the experimental data at different temperatures to the Arrhenius equation (Eq. 10), the activation energy $\left({E}_{a}\right)$ and pre-exponential factor (A) can be calculated.

10 $$k=Aexp\left(-\frac{{E}_{a}}{RT}\right).$$

Linearization of Eq. (10) above results in Eq. (11).

11 $$Ink=InA- \frac{{E}_{a}}{R} \times \frac{1}{T}.$$

A plot of $Ink$ against $\frac{1}{T}$ yields a slope = $-\frac{{E}_{a}}{R}$ and intercept $=$ $InA$.

Furthermore, because the esterification reaction process was carried out at different temperatures, the thermodynamic properties of OA esterification over 6TsOH-MBC catalyst, including enthalpy change ($\Delta H$), Gibbs free energy (($\Delta G$) and entropy change ($\Delta S$), were estimated using the following expressions^27–29^.

12 $$\Delta H= {E}_{a}-RT,$$

13 $$\Delta G= {E}_{a}+ RTIn\left(\frac{{K}_{B} \times T}{h \times A}\right),$$

14 $$\Delta S= \frac{\Delta H- \Delta G}{T},$$

where ${K}_{B} (Boltzman \; constant)=1.38 \times {10}^{11}\mathrm{ J} \; {\mathrm{K}}^{-1}$, $h (Plank \; constant)=6.626 \times {10}^{-23} \; \mathrm{ Js}$, T is absolute temperature (K) and R is the gas constant (8.314 J mol^−1^ K^−1^).

### Approval and compliance with regulation

This research work was formally authorized by the Academic and Research Unit, Afe Babalola University, Ado-Ekiti, Nigeria. Experimental research and field studies on collected eucalyptus tree bark comply with relevant institutional guidelines and legislation.

## Results and discussion

### FAME yields of the catalyst samples

**Figure 1:**
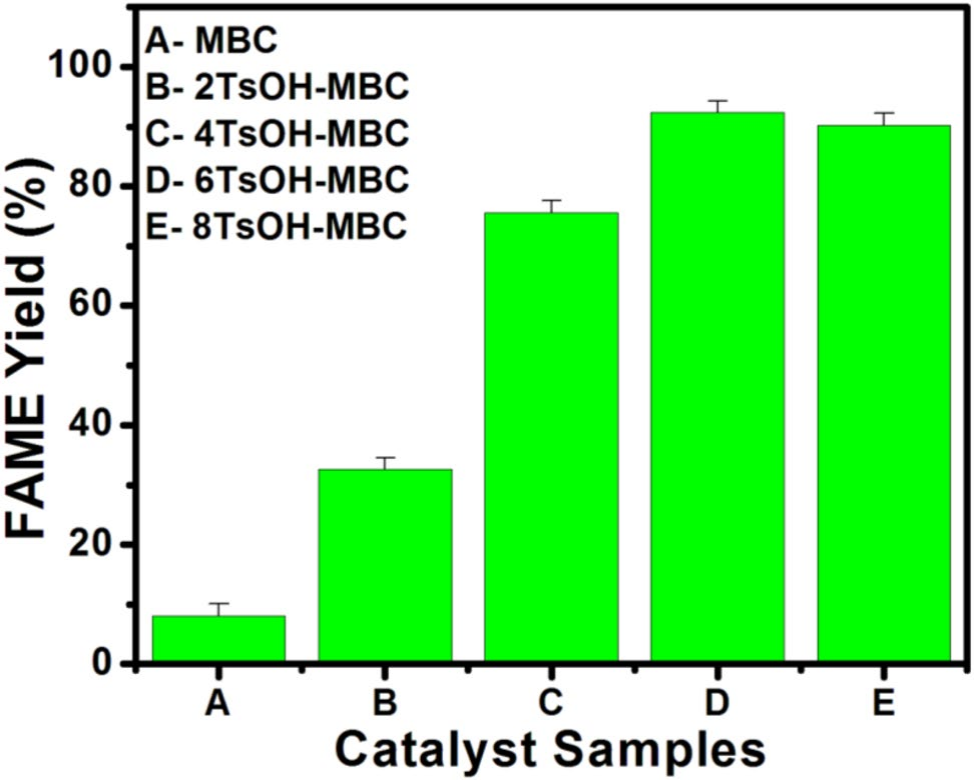
FAME content against the various catalyst samples used.

In this study, four samples of TsOH sulfonated MBC were synthesized, corresponding to the four different masses of TsOH used during the sulfonation process. TsOH-MBC catalysts were obtained in the following concentrations: 2TsOH-MBC, 4TsOH-MBC, 6TsOH-MBC and 8TsOH-MBC. To assess the activity of each catalyst sample, they were used to catalyze the esterification of oleic acid with methanol, and the values of FAME yields obtained were plotted against the different TsOH-MBC catalyst samples used, as shown in Fig. 1.

As shown in the results (Fig. 1), the MBC appeared to be ineffective for converting OA to FAME due to a lack of acidic sites on its surface, preventing the reaction from reaching equilibrium. However, when TsOH was loaded onto MBC, there was a significant increase in FAME yield with increasing the amount of the sulfonating agent used in the sulfonation process. The trend shown in Fig. 1 indicated an increase in FAME content from 32.6 to 92.3%, with a corresponding increase in TsOH mass from 2 to 6 g. When 8 g of TsOH was loaded on the same amount of MBC, the FAME yield decreased significantly. The possible reason for the decreasing of catalyst activity with increasing TsOH amount is the TsOH penetration through the MBC in various ways depending on the uptake level of catalyst support towards sulfonating agent. The TsOH was well dispersed on the MBC surface at low concentration of TsOH, but at higher concentration of TsOH, the acid molecules deposited on the surface of MBC and blocked the active sites^1,30^. These findings indicated that 6TsOH-MBC was the most effective of the prepared catalysts, so it was chosen for further investigation.

The sulfonation process, in general, introduces both sulfonic acid (–SO_3_H) and oxygenated acid (–COOH) sites. The –SO_3_H functional groups, according to Zhang et al.^31^, act as the primary catalytic sites, while the –COOH groups promote the inherent activity of the –SO_3_H, facilitating oleic acid esterification. Protonation of methanol molecules occurs infrequently during the esterification reaction due to the strong nature of the –SO_3_H group. When a weak acid group, such as –COOH, is present, the deprotonated form of –COOH may form a hydrogen bond with the –OH group in the methanol molecule, giving the oxygen in the alcohol molecule a negative charge. This negative charge, in turn, promotes the nucleophilicity of the alcohol molecule, increasing the reaction rate and conversion of oleic acid^32,33^.

### Analysis of the 6TsOH-MBC catalyst

The MBC and 6TsOH-MBC catalyst samples were characterized to gain insight into their physicochemical properties using SEM, FTIR, XRD, EDX, TGA, BET and acidity techniques as follows:

#### SEM analysis

**Figure 2:**
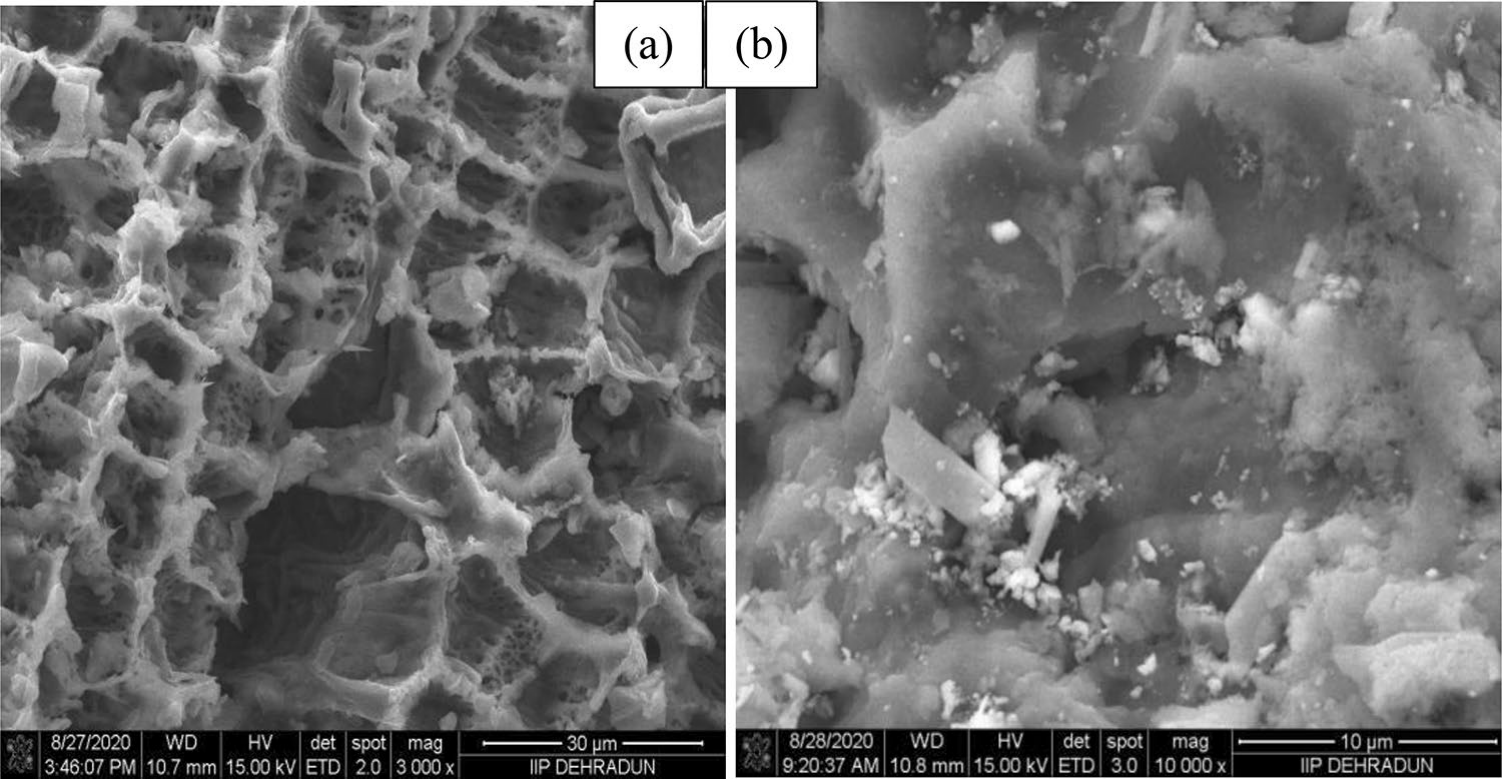
SEM images of (**a**) MBC and (**b**) 6TsOH-MBC catalyst.

**Figure 3:**
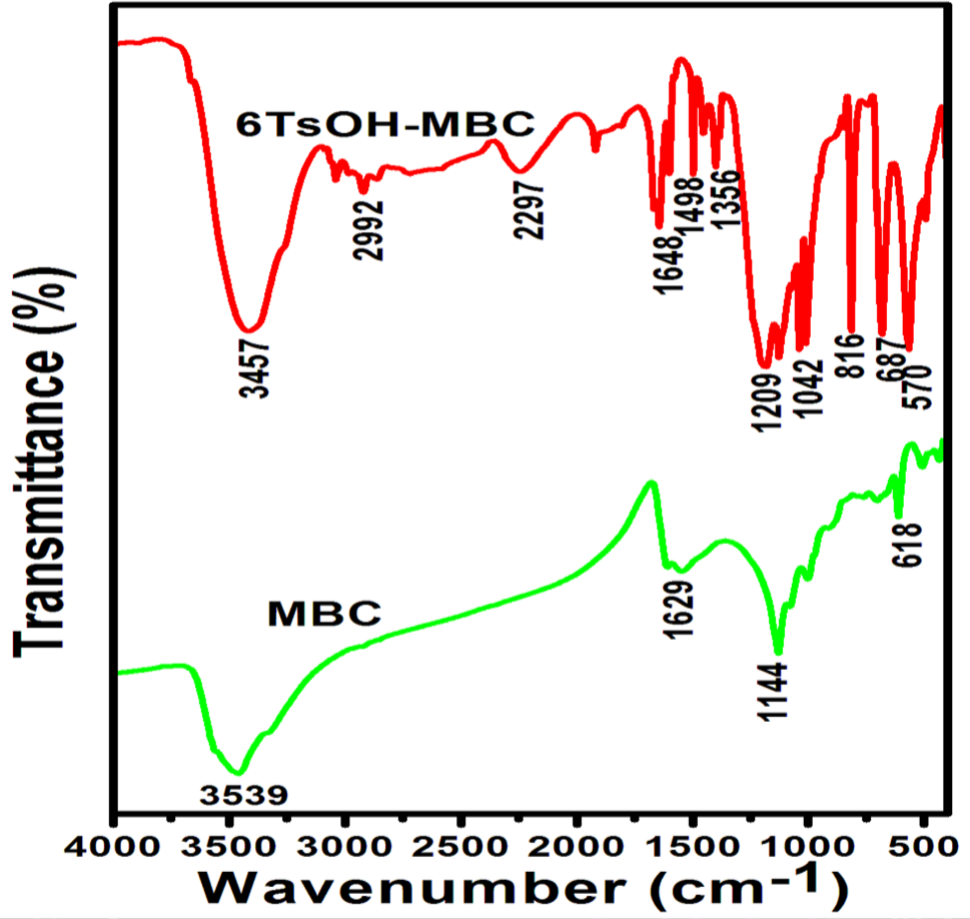
FTIR spectra of MCB and 6TsOH-MBC catalyst.

**Figure 4:**
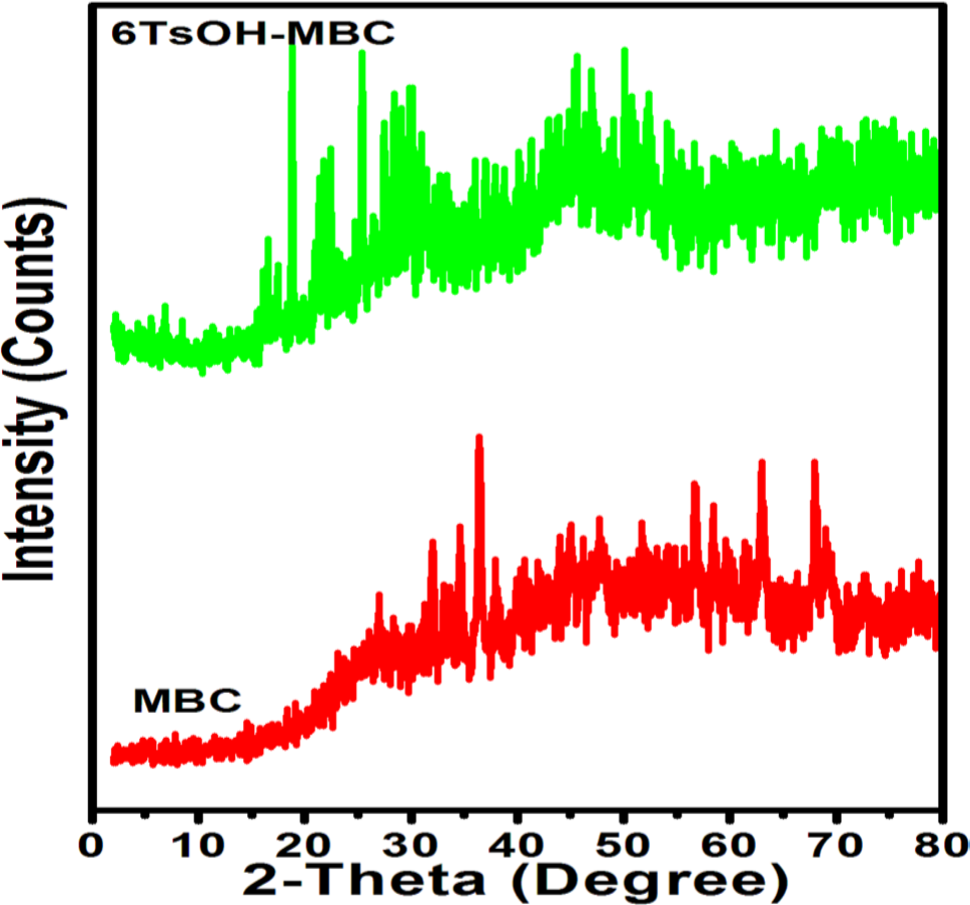
XRD patterns of MBC and 6TsOH-MBC.

Figure 2 depicts the morphologies of the MBC and 6TsOH-MBC catalyst samples. MBC had a sponge-like, rough and irregular surface structure, as shown in Fig. 2a, with many pores on its surface. However, as a result of the impregnation effect, the pores were no longer visible after TsOH was loaded on MBC (see Fig. 2b), indicating even dispersion of the SO_3_H group on the support. The FTIR results (see Fig. 3) corroborated this, revealing a transformation in the chemical structure of the catalyst support, with the formation of oxygen-containing functional groups such as SO_3_H, C–O–H, and OH groups observed. Furthermore, the SEM image revealed that the crystallinity of the 6TsOH-MBC was more pronounced than that of MBC, which was supported by the XRD pattern (Fig. 4), which revealed that the former had a more obvious amorphous carbon structure. It is worth noting that the cracks observed on the solid acid catalyst were most likely caused by partial collapse of the porous structure during the sulfonation process, as reported by Ngaosuwan et al.^1^. Nonetheless, the reacting molecules were able to easily penetrate through the available pores on the catalyst surface, as evidenced by the value of FAME content obtained when the 6TsOH-MBC catalyst was used for esterification, indicating the presence of active acidic sites on the catalyst surface^3^.

#### FTIR analysis

Figure 3 depicts FTIR data for the MBC and 6TsOH-MBC catalyst, which revealed several functional groups present on the analyzed samples. The wide peaks in the spectra of MBC and 6TsOH-MBC samples in the 3457–3539 cm^−1^ range were attributed to O–H stretching vibration, which could be traced back to hydrogen bonding in cellulose, phenolic compound and lignin^34^. On the MBC spectrum, the typical peaks were 1629 cm^−1^ (NH_2_ deformation), 1144 cm^−1^ (C–N stretching), and 618 cm^−1^ (C–O–H twisting)^35^. The sulfonation of eucalyptus biochar, on the other hand, resulted in a shift of the peak at 1629 cm^−1^ to 1648 cm^−1^ (NH_2_ deformation). Also, It led to formation of new peaks (see the spectrum of 6TsOH-MBC) at 2992 cm^−1^, 2297 cm^−1^, 1498 cm^−1^, 1356 cm^−1^, 1209 cm^−1^ and 1042 cm^−1^, which correponded to C–H stretching contained in methyl group, C=N stretching, CH_2_ deformation, S=O vibration reduction (sulfonic acid (SO_3_H) bond), C=S asymmetric stretching and S=O stretching vibration, respectively. The FTIR data obtained for sulfonated eucalyptus biochar were in agreement with those results reported for solid acid catalysts derived from palm kernel shell^11^, coffee residue^1^ and corncob^3^. When the FTIR data of activated biochar and sulfonated biochar catalyst were compared, it was possible to conclude that the sulfonic acid group was successfully inserted into the biochar, implying that acidic sites were active centers during the reaction process.

#### XRD analysis

X-ray diffractograms of the MBC and 6TsOH-MBC catalyst samples are shown in Fig. 4, with the former revealing some peaks that were attributed to ZnO phase at 2$\theta$ around 34°, 36° and 56° probably owing to the ZnCl_2_ used as a modifying agent. Ngaosuwan et al.^1^ reported similar finding whereby ZnO phase was detected in XRD diffractogram of ZnCl_2_-activated coffee residue biochar. However, a visible change in the XRD pattern was noticed after sulfonation of MBC sample, as seen in the pattern of 6TsOH-MBC. A peak at 2$\theta$ = 27.1° was noticed in the diffractogram of the solid acid catalyst, which corresponded to the (002) plane of amorphous carbon. This result was in line with the findings reported by Zhao et al.^36^ and Dechakhumwat et al.^3^ who synthesized sulfonated biochar catalysts from waste pomelo peel and corncob, respectively.

#### EDX analysis

As listed in Table 1, the MBC was made up of mainly C followed by O with traces of Zn and Cl owing to activating agent (ZnCl_2_) used for activated biochar, whereas 6TsOH-MBC catalyst contained C, O, Si, S, Cl and Zn. The presence of sulfur in the catalyst sample suggested successful impregnation of –SO_3_H group on the modified biochar. The composition of C and O in the 6TsOH-MBC catalyst decreased probably due to the sulfonation effect. In terms of C/O ratio, the sulfonated eucalyptus biochar catalyst exhibited larger ratio as compared to MBC which indicated that impregnation of TsOH on MBC increased oxygen-containing functional groups and contributed to the retention of oxygen content^13^. More so, it enhanced the hydrophilicity of the as-synthesized catalyst, thereby improving the reactant adsorption onto its surface^1,36^.

**Table 1:** EDX analysis for MBC and 6TsOH-MBC catalyst.

Element	Sample	
	MBC	6TsOH-MBC
Carbon (C)	70.58	66.88
Oxygen (O)	27.97	20.83
Chlorine (Cl)	0.23	1.63
Zinc (Zn)	1.23	3.44
Silicon (Si)	–	0.22
Sulphur (S)	–	7.02

#### Thermal stability analysis

**Figure 5:**
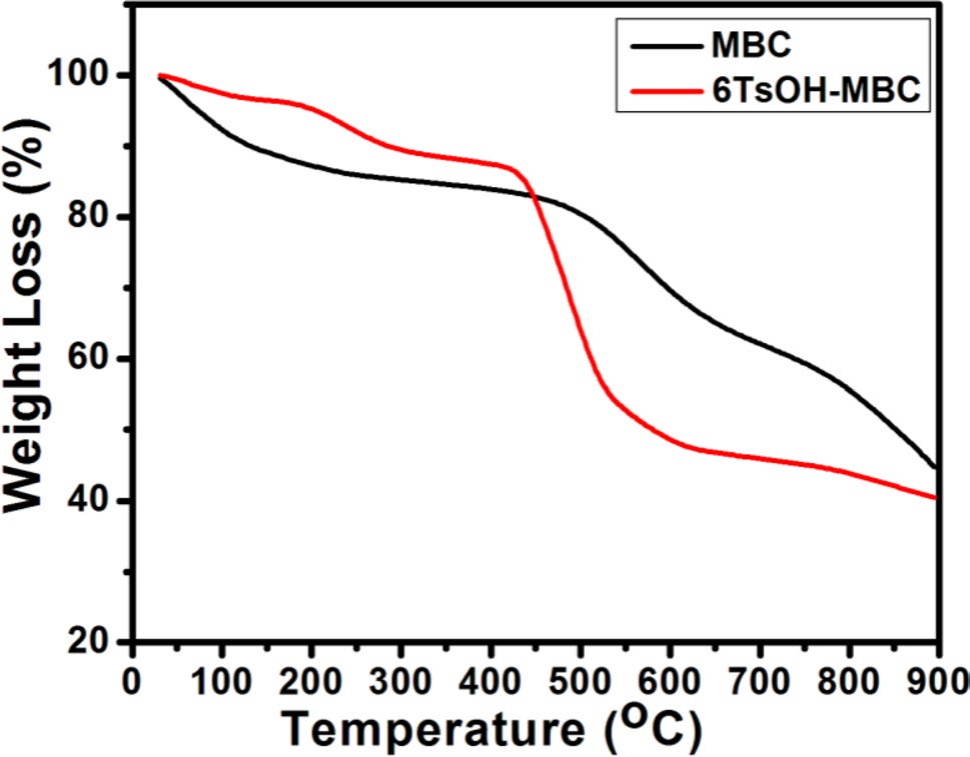
TGA of MBC and 6TsOH-MBC.

In a bid to investigate the thermal decomposition trends of the activated biochar and sulfonated biochar catalyst, TGA analysis was conducted on both samples. As evident in Fig. 5, the support and solid acid catalyst exhibited different decomposition trends owing to sulfonation effect. Two weight loss stages were observed in the TGA curve for MBC. However, 6TsOH-MBC catalyst exhibited three stages of mass loss, as seen in Fig. 5, indicating that the latter had better thermal stability than the former. The first stage of decomposition of the heterogeneous acid catalyst occurred between 30 and 405 °C, which was as a result of the simultaneous removal of water molecules and decomposition of sulfonic acid (–SO_3_H groups)^11,36,37^. Since degradation of lignin usually occurs at 200–500 °C^38^, the second degradation stage, which took place from 405 to 606 °C, suggested that sulfonation led to complete decomposition of lignin as well as complete removal of hemicellulose and cellulose^39^. However, the last stage of weight loss by the sulfonated biochar catalyst occurred above 600 °C which suggested that the 6TsOH-MBC was thermally stable due to carbon moiety^11,40^, as reaffirmed by the FTIR and EDX analyses.

#### Textural characteristics and the acidity

**Figure 6:**
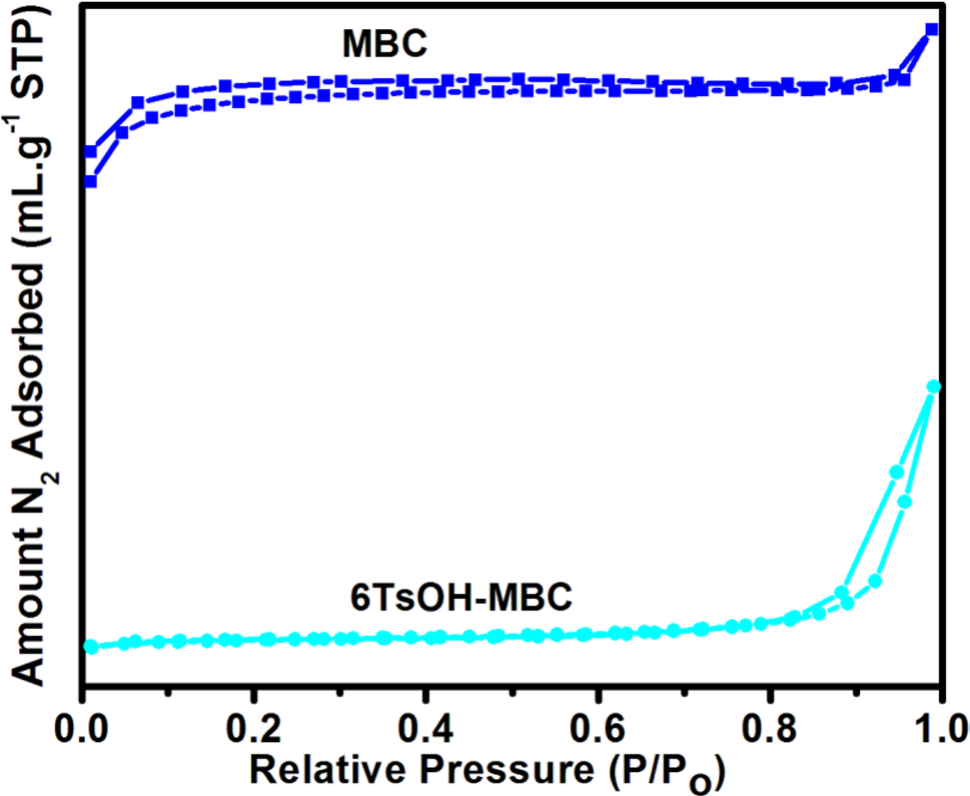
N_2_ adsorption isotherms for the MBC and 6TsOH-MBC.

The N_2_ adsorption isotherms of the MBC and 6TsOH-MBC samples are shown in Fig. 6, with the former exhibiting a type II isotherm and the latter exhibiting a type IV isotherm (mesoporous material). Table 2 presented the textural characteristics of the MBC and 6TsOH-MBC samples. The BET specific surface area, total pore volume and average pore size diameter reduced after sulfonation process. Similarly, Zhang et al.^41^ reported a significant decrease in surface area during TsOH impregnation on glucose. The possibility of –SO_3_H multilayer formation on the MBC surface during sulfonation could not be ruled out. The partial blockage of the pores by multilayer dispersion of –SO_3_H reduced the specific surface area^30^, which corresponded to the SEM images of the sulfonated biochar catalyst (see Fig. 2).

**Table 2:** Textural characteristics and acidity of MBC and 6TsOH-MBC catalyst.

Parameter	Sample	
	MBC	6TsOH-MBC catalyst
BET surface area (m^2^/g)	217.3	30.7
Total pore volume (cm^3^/g)	0.21	0.24
Average pore diameter (nm)	49.9	24.6
Acidity (mmol of H^+^/g cat)	0.02 $\pm$ 0.053	0.469 $\pm$ 0.081

However, considering the result presented in Table 2, the low surface area of the 6TsOH-MBC catalyst did not signify low activity. The good activity exhibited by the sulfonated catalyst during esterification reaction was certainly due to the presence of acidic sites, considering that the acid density of the MBC was relatively low, 0.02 $\pm$ 0.053 mmol of H^+^/g cat. According to Refaat^42^, catalytic performance is closely related to the acidic sites density and hence the solid catalyst with higher acid density is expected to show better activity during reaction. Thus, this suggested the reason why 6TsOH-MBC catalyst exhibited good performance in esterification of OA.

### Esterification reaction condition

#### Influence of catalyst dosage

**Figure 7:**
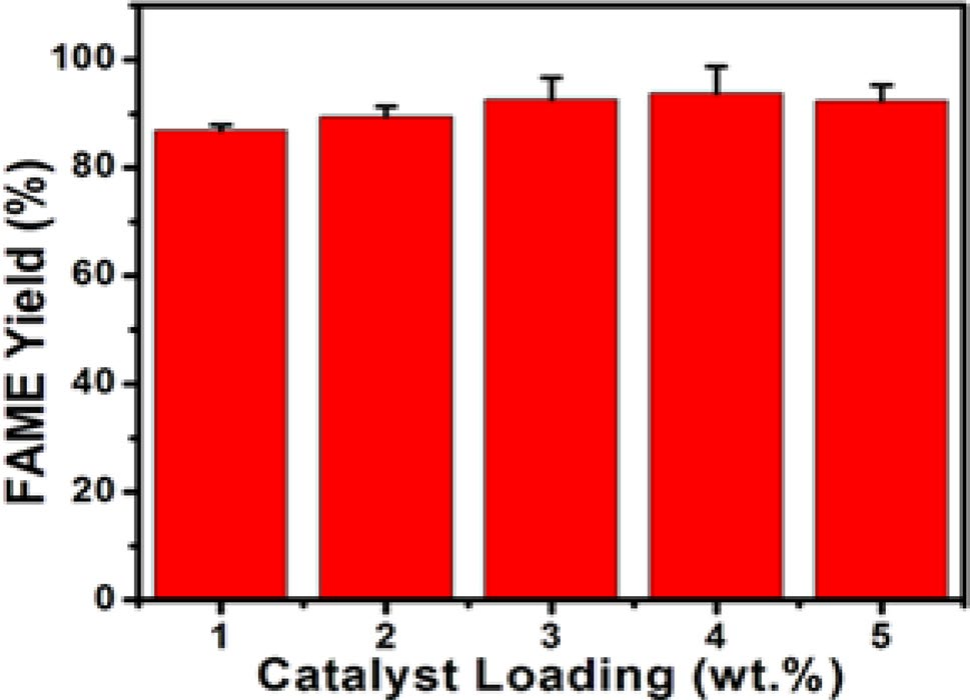
Influence of catalyst dosage on esterification process at 65 °C reaction temperature, 5 h reaction time, and 8:1 molar ratio of methanol to OA.

The results obtained during the investigation of catalyst loading influence on the esterification of OA at 65 °C for 5 h with an 8:1 methanol/OA molar ratio using a 1–5 wt% 6TsOH-MBC catalyst dosage are shown in Fig. 7. The FAME content increased with the amount of 6TsOH-MBC catalyst used up to 4.0 wt% and then decreased as the catalyst amount increased to 5.0 wt% due to the increasing viscosity of the reaction mixture, which inhibited the reaction^30,43^. This finding suggested that increasing the catalyst loading could not guarantee increase in FAME yield but raises the overall production cost of biodiesel. As a result, the optimum catalyst dosage of 4.0 wt% was chosen and used for the subsequent esterification reaction studies.

#### Influence of reaction temperature and time

**Figure 8:**
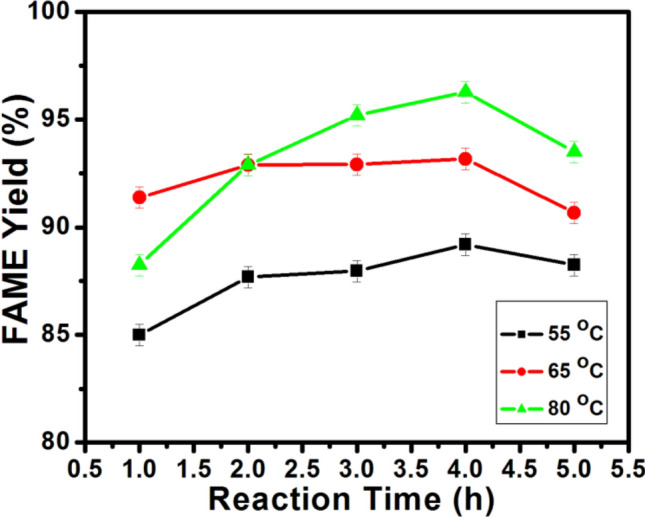
The influence of reaction temperature on esterification process: The reaction conditions: 8:1 methanol/OA molar ratio, 4.0 wt% catalyst loading and reaction duration of 5 h.

The influence of reaction temperature on the esterification process at different reaction durations (1–5 h) is illustrated in Fig. 8. The reaction tmperature was investigated at 55, 65 and 80 °C. The esterification reaction conducted at 80 °C for 5 h gave the maximum FAME content of 96.28%, suggesting that increasing the reaction temperature could raise the molecular collision and lower activation barrier, thereby enhancing the rate of reaction^30,44^.

### Reusability of 6TsOH-MBC catalyst

**Figure 9:**
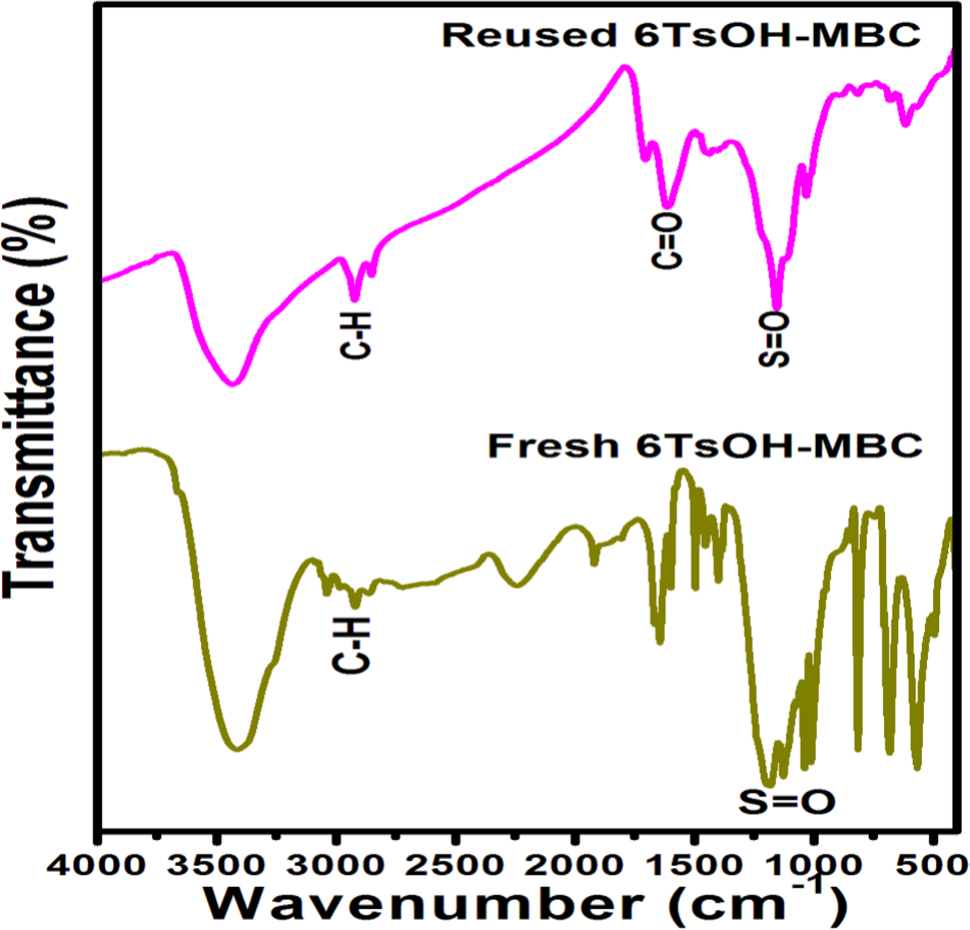
FTIR spectra of fresh and reused 6TsOH-MBC catalyst.

After each experiment, the spent catalyst was collected, washed with n-hexane to remove adhered fatty acid molecules, dried overnight in an oven at 100 °C, and then reused for another esterification reaction. FAME yields of 80.6%, 62.1%, and 38.7% were obtained for three consecutive cycles of oleic acid esterification at 80 °C for 5 h with a methanol/OA molar ratio of 8:1 and a 4.0 wt% regenerated 6TsOH-MBC dosage. This observation was attributed to a decline in the number of acidic sites owing to poisoning of the catalyst surface by fatty acid molecules and washing during catalyst regeneration process, which reduced the FAME content^1,43^. This is supported by the FTIR analysis of the regenerated catalyst (Fig. 9), which revealed a peak associated with C=O (ester) stretching from fatty acid methyl ester^45,46^. Furthermore, the intensity of peaks (1200 cm^−1^ and 1040 cm^−1^) associated with the sulfonic group (–SO_3_H) decreased, confirming the loss of acidic sites. This result suggested leaching of the active ingredient (SO_3_H), so a leaching analysis was performed by suspending the solid acid catalyst in methanol and heating the suspension at 55 °C for 2 h. Following that, the methanol was recovered by filtration and used for esterification of OA at 80 °C for 5 h with a methanol/OA molar ratio of 8:1 and a catalyst loading of 4.0 wt%. When the used methanol reacted with OA, a FAME yield of 10.07% was obtained, indicating that leaching of the 6TsOH-MBC occurred. As a result, the decrease in FAME content observed during several runs of the reaction process was caused by catalyst leaching. The observed trend was corroborated by the value of acidity of the 6TsOH-MBC which decreased to 0.108 $\pm$ 0.072 mmol of H^+^/g cat from 0.469 $\pm$ 0.053 mmol of H^+^/g cat after the third reaction cycle, as a result of possible leaching of –SO_3_H groups during esterification reaction. Similarly, a significant drop in acidity of H_2_SO_4_ sulfonated murumuru kernel shell biochar from 4.2 mmol of H^+^/g cat to 2.20 mmol of H^+^/g cat was recorded after the catalyst was reused for the fourth reaction cycle^32^.

### Esterification kinetics and thermodynamic studies

#### Kinetics studies

**Figure 10:**
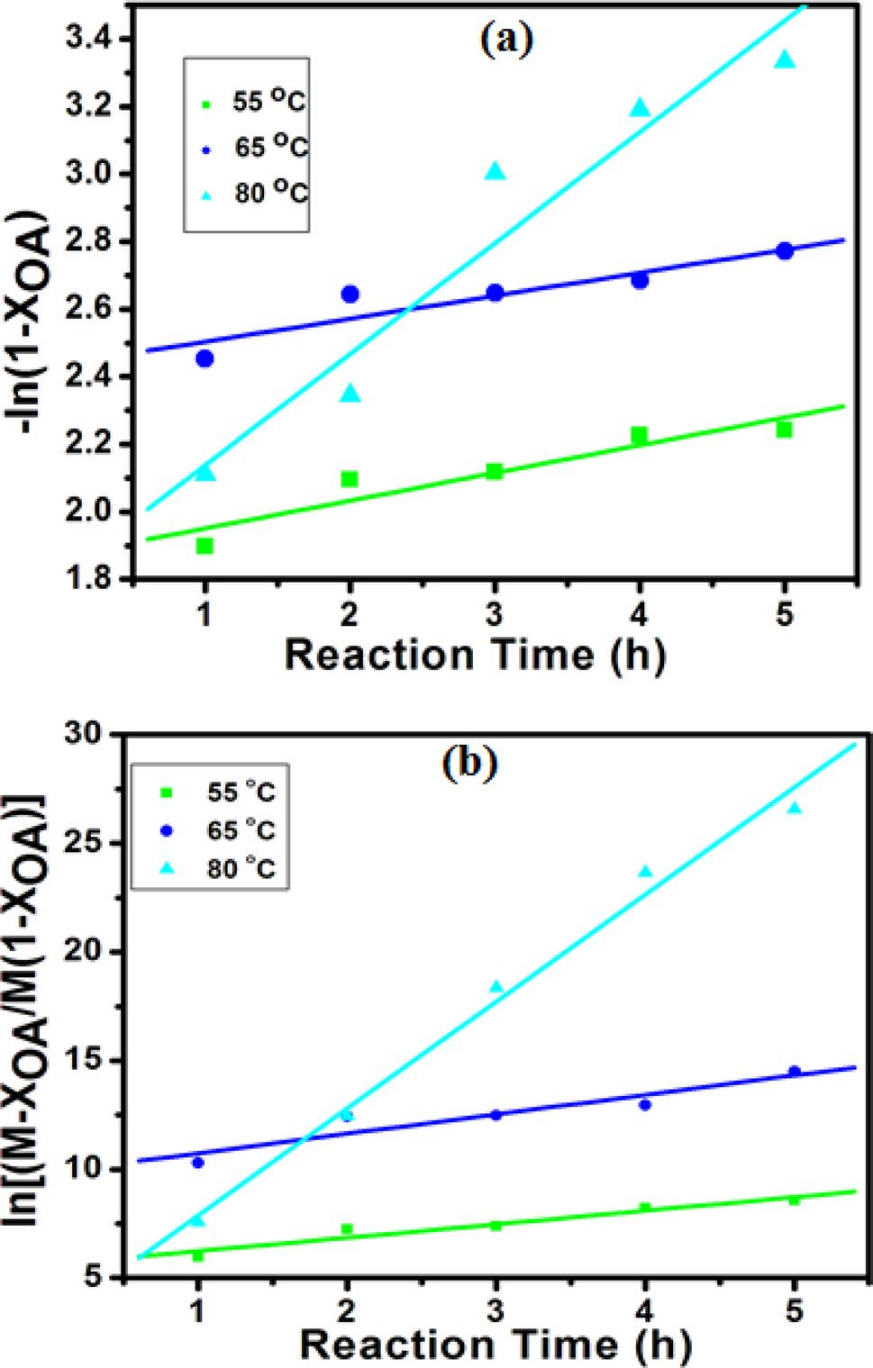
Fitting the kinetic models (**a**) PFO (**b**) second-order models for esterification of OA. The reaction condition: 4.0 wt% catalyst loading; methanol/OA molar ratio of 8:1 and 5 h reaction time.

Figure 10a, which illustrates the linear plot of –$In(1 - {X}_{OA})$ against reaction time at different reaction temperatures (PFO), facilitates the estimation of reaction rate constant (${k}_{1}$) from the slope of the plot and coefficient of determination (${R}^{2}$). A linear plot with high ${R}^{2}$ value indicated that such a kinetic model equation is suitable in predicting the catalytic reaction process^30,47^. In this study, however, PFO did not adequately predict the esterification of OA owing to low ${R}^{2}$ values (0.8465–0.9334) for all reaction temperatures, as seen in Table 3.

**Table 3:** Reaction temperatures, rate constants and ${R}^{2}$ values of oleic acid esterification over 6TsOH-MBC catalyst.

Reaction temperature (°C)	PFO		Second-order	
	${k}_{1}$ (h^−1^)	${R}^{2}$	${k}_{2}$ (mL mol^−1^ h^−1^)	${R}^{2}$
55	0.0819	0.8822	0.0282	0.9363
65	0.0681	0.8465	0.0406	0.8798
80	0.3291	0.9334	0.2233	0.9895

In an attempt to establish the appropriate kinetic equation for predicting the esterification of OA over 6TsOH-MBC catalyst, the second-order equation was employed and the constants (${k}_{2}$ and ${R}^{2}$) were estimated at different temperatures from the plot shown in Fig. 10b. As contained in Table 3, the ${R}^{2}$ values were determined to be in the range 0.8798–0.9895 and were greater than those of the PFO equation. Thus, the esterification reaction using 6TsOH-MBC catalyst appeared to be second-order with respect to oleic acid and methanol. Moreover, as also seen in Table 3, the reaction rate constants increased with increasing reaction temperature. The presumed reason was that when the reaction temperature was increased, the collision of molecules was enhanced, which led to decline in mass transfer limitation thus rate of reaction increased^30,48^.

#### Thermodynamic studies

**Figure 11:**
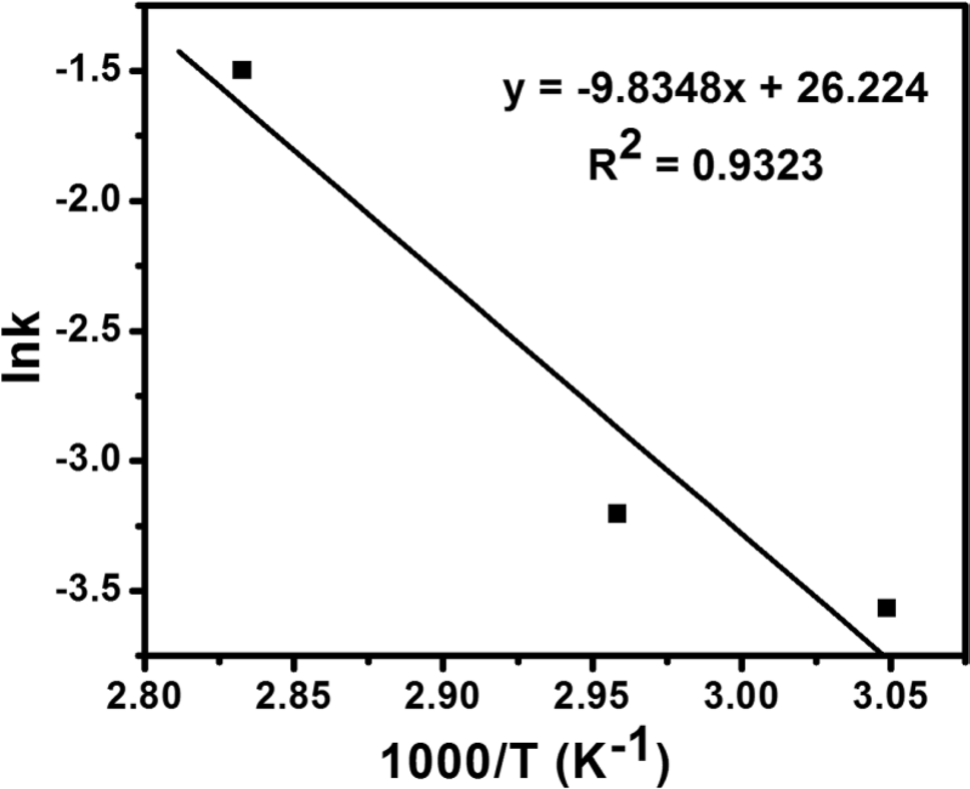
Plot of $Ink$ against 1/T (second-order model) for esterification of OA over 6TsOH-MBC catalyst.

Because the esterification reaction process was studied at various temperatures, estimating activation energy was critical in studying the effect of temperature on reaction rate^49,50^. The slope and intercept of the plot of $Ink$ against 1/T (Fig. 11) were used to estimate the values of ${E}_{a}$ and A, which were then used to calculate the reaction thermodynamic parameters such as $\Delta H$, $\Delta G$ and $\Delta S$. Their estimated values are presented in Table 4. The values of ${E}_{a}$ and A were found to be 81.77 kJ mol^−1^ and 2.45 $\times$ 10^11^ h^−1^, respectively. The activation energy for the esterification of OA over 6TsOH-MBC increased the reaction rate, which was consistent with the value range (33.6–84 kJ mol^−1^) for esterification of fatty acid using solid acid catalyst^51^, indicating that the catalytic reaction was much easier to carry out using SO_3_H-functionalized biochar catalyst as compared to other solid acid catalysts.

**Table 4:** Thermodynamic parameters for esterification of OA over 6TsOH-MBC catalyst.

Temperature (°C)	$\Delta H$ (kJ mol^−1^)	$\Delta G$ (kJ mol^−1^)	$\Delta S$ (J mol^−1 ^K^−1^)	${E}_{a}$ (kJ mol^−1^)	A (h^−1^)	${R}^{2}$
55	79.04	32.94	140.55	81.77	2.45 $\times$ 10^11^	0.9323
65	78.96	33.02	135.91			
80	78.83	33.14	129.44			

The values of thermodynamic parameters for esterification of OA with methanol over 6TsOH-MBC catalyst are also presented in Table 4. The positive values of $\Delta H$ suggested that the catalytic reaction process was endothermic. The endothermic and nonspontaneous esterification process was confirmed because the $\Delta G$ value for each temperature was positive^48,52^. Moreover, the increase in values of $\Delta G$ as the temperature increased suggested an enhanced reaction rate at higher temperature^28^. Entropy, which measures the degree of disorderliness in a reaction system, was also calculated. Table 4 shows that the values of entropy change $(\Delta S)$ for the three reaction temperatures investigated were all positive, indicating an increase in irregularity and randomness at the solid–liquid interface during the esterification reaction. Based on these findings, it is possible to conclude that the 6TsOH-MBC catalyst demonstrated a faster reaction rate, moderate activation energy and mass transfer resistance during the esterification reaction process.

## Conclusions

This study found that the SO_3_H-loaded eucalyptus biochar catalyst was effective for the esterification of OA to produce methyl ester. The well-developed porous structure, dominance of active acidic sites and well dispersed TsOH, as validated by SEM, FTIR, acidity analysis and EDX, contributed to the 6TsOH-MBC catalyst performance in the esterification process. At 80 °C for 5 h with an 8:1 methanol/OA molar ratio and 4.0 wt% catalyst loading, a maximum FAME content of 96.28% was obtained. Analysis of kinetic data using two different kinetic models showed that the esterification of OA over 6TsOH-MBC was second-order with respect to each reactant. Studies on thermodynamic behaviour of the reaction indicated that the esterification of OA with methanol over 6TsOH-MBC catalyst was nonspontaneous and endothermic. These findings confirmed that the SO_3_H functionalized eucalyptus biochar catalyst could be widely used for large-scale sustainable biodiesel production.
